# Use of rivaroxaban in sickle cell disease and venous thromboembolism: A case report

**DOI:** 10.1097/MD.0000000000027988

**Published:** 2021-12-23

**Authors:** Waail Rozi, Elrazi Awadelkarim Hamid Ali, Abdulrahman F. Al-Mashdali, Mohamed Abdelrazek, Mohamed A. Yassin

**Affiliations:** a Internal Medicine, Hamad Medical Corporation, Doha, Qatar; b Radiology Department, Hamad Medical Corporation, Doha, Qatar; c Hematology and Oncology, Hamad General Hospital, Doha, Qatar.

**Keywords:** direct oral anticoagulant, pulmonary embolism, rivaroxaban, sickle cell disease, venous thromboembolism

## Abstract

**Introduction:**

Sickle cell disease (SCD) is a hematological disorder characterized by sickling of red blood cells. Patients are at increased risk of venous thromboembolism. There are no guidelines for the management of venous thromboembolism in sickle cell disease specifically in terms of the anticoagulant of choice.

**Patient concerns:**

Here, we report a case of a 30-year-old lady with past medical history of sickle cell disease who came with chest pain and shortness of breath.

**Diagnosis:**

We found that she has bilateral pulmonary embolism (PE).

**Intervention:**

She was started on rivaroxaban.

**Outcome:**

The patient was followed for 18 months, she did not suffer from recurrence of PE, and she did not develop any complications related to rivaroxaban.

**Conclusion:**

We concluded that rivaroxaban is effective in treating PE in sicklers and also it is safe.

## 1 Introduction

Sickle cell disease is an inherited hematological disease. The normal hemoglobin is formed of 2 alpha and 2 beta units and called hemoglobin A. In patients with sickle cell anemia who is having abnormal gene from both parents, beta unit synthesis is defected and leads to formation of hemoglobin S which is prone to sickling under certain circumstances.^[1]^ Sickling happens because of mutation in beta globin formation in which valine is replacing glutamic acid. Prevalence sickle cell disease (SCD) is more common in citizens of Middle Eastern, sub-Saharan African, and other Mediterranean countries.^[2]^ Patient with sickle cell anemia suffers from various complications especially recurrent hemolysis and vaso-occlusive crisis stroke and deafness.^[3]^ One of the most recognizable complication is venous thromboembolism (VTE), either deep vein thrombosis or pulmonary embolism (PE). VTE incidence and recurrence is SCD is higher than general population and around 10% of total patients would develop VTE before the age of 40.^[3]^ Long-term treatment of VTE in sickle cell patient is not well studied despite high recurrence rate and the increase in the mortality and morbidity incidence.^[4]^

## 2 Case presentation

We present the case of a 30-year-old lady of Arabic decent, who presented to emergency department with one day history of chest pain and shortness of breath. She is a known case of sickle cell disease and on deferasirox for iron overload and folic acid. She has no previous history of venous thromboembolic disease and had no recent operations.

**Figure 1:**
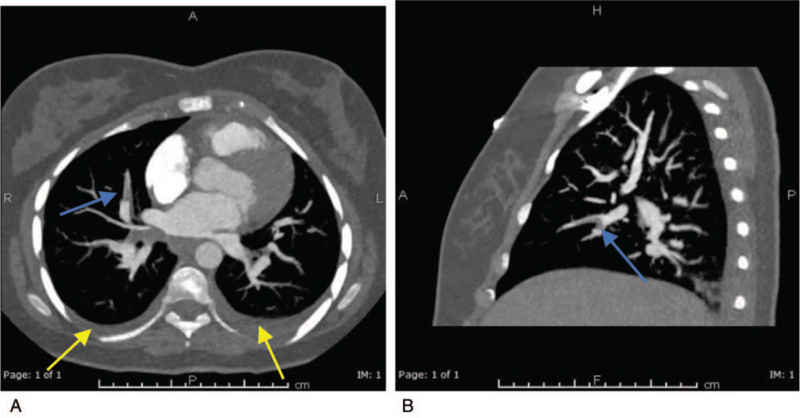
Axial (A) and sagittal (B) minimum intensity projections (MIP) of CT scan of the chest with intravenous contrast administration for pulmonary embolism showing filling defect in the right middle lobe segmental branch (Blue Arrow in A&B) denoting pulmonary embolism. There is also mild bilateral pleural effusion (yellow arrows in B).

On examination, she was tachypneic, tachycardic, and desaturating in which she required O_2_ therapy through nasal canula to maintain saturation >94%. Physical examination was unremarkable. Chest x-ray was normal. She was labeled as case of acute chest syndrome and exchange transfusion was done. Despite that, her condition did not improve, and she was persistently tachypneic, tachycardic, and requiring oxygen to maintain saturation. Echo was done to rule out cardiac issue and showed only high pulmonary artery pressure which raised the concerns of possibility of PE. CT pulmonary angiography was done and showed linear filling defect noted at the bifurcation of superior and inferior lingular segment of the left pulmonary artery, filling defect at truncus anterior branch of right upper pulmonary artery, and filling defect at the right middle lobe segmental branch confirming the diagnosis of bilateral PE (Fig. 1). She was started on rivaroxaban 15 mg twice daily for 21 days then 20 mg daily.

Her symptoms resolved and she was kept on rivaroxaban. The patient is on regular follow up, 16 months after the start of rivaroxaban, she did not have any major or minor bleeding and no recurrence of VTE.

## 3 Discussion

SCD is a terminology used to represent individuals with hemoglobin S . It is an autosomal recessive disorder that could present in 3 possibilities. First, patients may have both copies of the gene mutated, and the hemoglobin forms sickle shaped cells when exposed to hypo-oxygenation conditions. This form is called SCA. Second, heterozygous state; when the individual has a single beta gene mutation and the other gene is normal; these people are considered benign carriers, and this form is called sickle cell trait. These types of patients usually have less symptoms and complications of sickle cell disease. The last possibility is that patient may have single mutation in beta gene and another abnormal hemoglobin gene of a different type, these include hemoglobin SC; sickle-beta thalassemia; hemoglobin S/Lepore; and others.^[5]^

Direct oral anticoagulants (DOACs), including rivaroxaban and apixaban are oral anticoagulants used for the prevention of thrombosis in several cardiovascular indications like VTE and PE.^[6]^ Bleeding is the major complication of oral anticoagulation; however, when compared to vitamin K antagonist (VKAs), DOACs use was associated with less major bleeding events, specifically intracranial bleeding as demonstrated in a meta-analysis of 12 randomized controlled trials comparing DOACs to VKAs among patients with venous thromboembolism or atrial fibrillation.^[7]^

Sickle cell disease is an significant cause of morbidity and mortality worldwide, causing harm and dysfunction in several organs.^[8]^ Patients with SCD are at high risk for thrombosis. All of the elements of Virchow triad (stasis of blood flow, hypercoagulability, and vascular wall injury) have been implicated in the pathogenesis of venous thromboses patients with sickle cell diseases.^[9]^ Around 10% of sickle cell disease patient suffer from VTE before the age of 40. Risk of death is triple in these patient when comparing to general population.^[10]^ Jacques-Robert Christen et al, found in their study that bleeding incident was less common in DOAC patient in comparing to patient taking VKAs. Also, there was no report major bleeding during the use of DOAC. In another retrospective study, Bhat et al reported 7 patients on rivaroxaban. Only 2 patients out of the 7 had major events. First patient had cerebral hemorrhage and the second patient had acute recurrent deep vein thrombosis. Nevertheless, they found that using DOAC leads to less frequent monitoring and hospital visit.^[11]^

Upon reviewing the literature, there were a few articles have discussed the benefits and risks of using DOAC and comparing DOAC to VKA in patient with SCD. We did not find a meta-analysis or systematic review discussing the safety and efficacy of using DOAC in VTE in patient SCD

Despite the high mortality and morbidity related to VTE in SCD patient, the evidence guiding the management of VTE in SCD specifically in terms of the anticoagulant of choice is not well studied.

Patel et al found that the use of DOACs, including rivaroxaban in comparison to other anticoagulation for the treatment of VTE in SCD was associated with better safety profile in terms of significant reduction in major bleeding events.^[12]^ Similarly, Roberts et al reported that the use of DOACs for VTE treatment in SCD compared to VKAs resulted in similar effectiveness in terms of VTE recurrence, but the use of DOACs was associated with a similar safety in comparison to VKAs in contrary to the results reported by Patel et al in their retrospective study.^[13]^

## 4 Conclusion

SCD is associated with many complications and one of them is venous thromboembolism which should be suspected in patient presenting with chest symptoms.

The optimal medication and the proper duration of using it is not well encountered till the date. Although, using of DOAC seems to be safe and effective depending on the current data and being used more frequently in the practice. Large and comparative prospective studies are mandatory to establish evidence-based recommendation in the best way to treat and follow VTE in Sickle patients.

## Acknowledgments

We wish to thank Qatar National Library and Internal Medicine Residency Program Director Dr Dabia Al-mohanadi and Deputy Program Director Dr Essa Abuhelaiqa in Hamad Medical Corporation for the scientific support.

## Author contributions

**Conceptualization:** Elrazi Awadelkarim Hamid Ali, Mohamed A. Yassin.

**Data curation:** Waail Rozi.

**Investigation:** Mohamed Abdelrazek.

**Methodology:** Waail Rozi, Elrazi Awadelkarim Hamid Ali, Abdulrahman F. Al-Mashdali.

**Project administration:** Mohamed A. Yassin.

**Resources:** Mohamed A. Yassin.

**Supervision:** Mohamed A. Yassin.

**Visualization:** Mohamed A. Yassin.

**Writing – original draft:** Waail Rozi, Elrazi Awadelkarim Hamid Ali.

**Writing – review & editing:** Waail Rozi, Elrazi Awadelkarim Hamid Ali, Abdulrahman F. Al-Mashdali, Mohamed Abdelrazek, Mohamed A. Yassin.
